# Steroidal saponins from the roots of *Solanum sisymbriifolium* Lam. (Solanaceae) have inhibitory activity against dengue virus and yellow fever virus

**DOI:** 10.1590/1414-431X2020e10240

**Published:** 2021-05-17

**Authors:** G.G. Figueiredo, O.A. Coronel, A.C. Trabuco, D.E. Bazán, R.R. Russo, N.L. Alvarenga, V.H. Aquino

**Affiliations:** 1 Laboratório de Virologia, Departamento de Análises Clínicas, Toxicológicas e Bromatológicas, Faculdade de Ciências Farmacêuticas de Ribeirão Preto, Universidade de São Paulo, Ribeirão Preto, SP, Brasil; 2 Department of Phytochemistry, Faculty of Chemical Sciences, National University of Asuncion, San Lorenzo, Paraguay

**Keywords:** Dengue virus, Yellow fever virus, Solanum sisymbriifolium, Antiviral, Virucidal

## Abstract

Dengue is the most important arthropod-borne viral disease worldwide. Infection with any of the four dengue virus (DENV) serotypes can be asymptomatic or lead to disease with clinical symptoms ranging from undifferentiated and self-limiting fever to severe dengue disease, which can be fatal in some cases. Currently, no specific antiviral compound is available for treating DENV. The aim of this study was to identify compounds in plants from Paraguayan folk medicine with inhibitory effects against DENV. We found high virucidal activity (50% maximal effective concentration (EC_50_) value of 24.97 µg/mL) against DENV-2 in the ethanolic extract of the roots of *Solanum sisymbriifolium* Lam. (Solanaceae) without an evident cytotoxic effect on Vero E6 cells. Three saponins isolated from the root extract showed virucidal effects (EC_50_ values ranging from 24.9 to 35.1 µg/mL) against DENV-2. Additionally, the saponins showed inhibitory activity against yellow fever virus (EC_50_ values ranging from 126 to 302.6 µg/mL), the prototype virus of the *Flavivirus* genus, suggesting that they may also be effective against other members of this genus. Consequently, these saponins may be lead compounds for the development of antiviral agents.

## Introduction

Dengue is the most important arthropod-borne viral disease, affecting approximately 390 million people per year, of which 96 million manifest diverse severity in the clinic (1). An estimated 3.9 billion people in 128 countries are at risk of infection with dengue virus (2). Dengue virus (DENV), which includes four distinct serotypes (DENV-1, DENV-2, DENV-3, and DENV-4), belongs to the *Flavivirus* genus, Flaviviridae family. Infection with any of the four DENV serotypes can be asymptomatic or lead to disease with clinical symptoms ranging from an undifferentiated and self-limiting fever to severe dengue disease, which is mostly characterized by plasma leakage and hypovolemic shock that can be fatal in some cases (3). A vaccine (Dengvaxia, Sanofi-Pasteur, France) against DENV infection received its first marketing authorization in late 2015 and is currently available in several Asian and Latin American countries. However, the World Health Organization (WHO) has recently recommended the administration of Dengvaxia only to individuals who have been previously infected with wild DENV due to the increased risk of hospitalization (and severe disease) for vaccinated subjects who are naive to wild DENV infection prior to vaccination (4). Although several promising molecules have shown potential anti-DENV effects (5–7), no specific antiviral compound is available against this virus. Therefore, the identification of compounds with an inhibitory effect against DENV is of great interest.

Yellow fever virus (YFV) is the causative agent of a severe acute hemorrhagic fever with a high mortality rate. An effective vaccine against yellow fever has been available for almost 70 years and is responsible for a significant reduction in the occurrence of the disease worldwide; however, approximately 200,000 cases of yellow fever and 30,000 deaths occur per year, with more than 90% of clinical cases occurring in Africa (8,9). No specific antiviral drugs are available for yellow fever treatment.

Plants used in traditional medicine constitute important sources of biologically active compounds. Paraguay has an ancient tradition of using medicinal plants, which are promising candidates for the isolation of useful substances capable of being potential lead compounds from which new therapeutics may be developed. Plants of the Solanaceae family have been used in many countries because of their medicinal properties, including those used in Ayurveda and Chinese traditional medicine (10). Most genera of the family have great importance either as food or as sources of medicinal substances that have been used today, such as hyoscyamine and scopolamine (11). The *Solanum* genus is probably one of the most important in the Solanaceae family with approximately 2000 species distributed worldwide. The members of this family have antiviral activity, making them candidates for novel antiviral molecules (12). One of the plants of this family used in Paraguayan folk medicine is *Solanum sisymbriifolium* (Solanaceae), a viscoid and very prickly erect shrub found in eastern Paraguay, known as *ãuatí pytâ* in Guarani, the indigenous language still spoken in Paraguay, or sticky nightshade in English. In Paraguay, the root of *S. sisymbriifolium* is used in the treatment of hypertension due to its hypotensive effect (13), which was confirmed by studies using both normo- and hypotensive rats (14,15). In Argentina, *S. sisymbriifolium* is used as a diuretic, analgesic, contraceptive, antisyphilitic, and hepatoprotective agent (16). The compound classes described in this species comprise alkaloids, lignans, steroids, and saponins (17–19).

In this study, we found anti-DENV activity in the ethanolic extract of *S. sisymbriifolium* roots, specifically three saponins. In addition, these saponins showed an inhibitory effect against YFV, the prototype virus of the *Flavivirus* genus, suggesting they may also be effective against other species in this genus.

## Material and Methods

### Plant material

*Solanum sisymbriifolium* Lam. (the entire plant) was collected in the Aguaity district, Cordillera department, Paraguay (latitude 25°26′12- S; longitude 57°4′24- W). The plant was identified at the Botany Department, Faculty of Chemical Sciences, National University of Asuncion, Paraguay. A voucher specimen (R. Degen 4126 *et* S. Kim, G. González, L. Britos) was deposited at the Herbarium of the Faculty of Chemical Sciences, National University of Asuncion, for indexation purposes.

### Preparation of the ethanolic extract of *S. sisymbriifolium* roots

*S. sisymbriifolium* roots (3 kg) were dried at room temperature (30°C) and then ground to a fine powder using a blade mill. The powder was distributed in 1000-mL beakers, and ethanol was added until all of the material was soaked. The material was then placed in an ultrasonic bath (ULTRASONS H-D, J.P. Selecta, Spain) for 30 min. The process was repeated three times. The material was soaked overnight and was then filtered through qualitative paper (GE Healthcare, Spain). The insoluble marc was extracted twice in the same manner. The filtered extracts were evaporated in a rotary evaporator (RVO 400 SD, Boeco, Germany). The insoluble marc was poured into a 3-L round-bottomed flask and refluxed with ethanol for 15 min. The liquid was filtered through qualitative paper, and the process was repeated twice. The extracts were evaporated in a rotary evaporator, and both extracts were mixed. The process yielded 29 g of crude extract (Phytochemistry Department in-house method, Paraguay).

### Isolation of steroidal saponins

Approximately 18 g of the crude extract was dispersed in water and extracted three times with 1-butanol. The butanolic extract was treated with ethyl acetate or diethyl ether until the crude saponins were precipitated. The precipitate was filtered through qualitative paper and air-dried (1.91 g were obtained, 10.6% yield). Subsequently, the sample was subjected to preparative column chromatography using silica gel (E. Merck KGaA, Germany, particle diameter of 0.063-0.2 mm) for the stationary phase and a mixture of hexane, ethyl acetate, and methanol at a ratio of 2:6:5 with 0.5% NH_4_OH for the mobile phase. After collecting the fractions, preparative thin layer chromatography (Macherey-Nagel GmbH & Co. KG, Germany) was performed to separate the individual compounds using silica gel for the stationary phase (20×20 cm with aluminum support, particle diameter <0.063 mm, and layer thickness of 0.5 cm). The mobile phase was the same as that for column chromatography. This final process was repeated many times to isolate enough material. The entire fractionation finally yielded saponins S1 (105 mg), S2 (115 mg), and S3 (207 mg) (Phytochemistry Department in-house method). Saponin S2 was identified by spectroscopic analysis using a Xevo TQD triple-quadrupole mass spectrometer (Waters Corporation, USA) with electrospray ionization in the negative mode for its mass spectrum. The 1H-NMR spectrum was recorded using a Bruker Advance III 400 (Bruker GmbH, Germany) at 400 MHz. Deuterated pyridine was used as the solvent.

### Solubilization of the root extract and saponins

The ethanolic extract of *S. sisymbriifolium* root (250 mg) was dissolved in 5 mL of ethanol by subsequent stirring and cooling cycles over 7 days. The supernatant containing soluble compounds was obtained by centrifugation at 2000 *g* for 5 min at 22°C. Saponins S1, S2, and S3 were dissolved in an aqueous solution containing 1% dimethyl sulfoxide (DMSO) to final concentrations of 8.1, 8.3, and 3.4 mg/mL, respectively.

### Cell lines and virus strain

Vero E6 cells were grown at 37°C in 24-well plates (2×10^5^ cells/mL) with L-15 medium (Leibovitz) (Cultlab, Brazil) supplemented with 10% inactivated fetal bovine serum (FBS) and 1% penicillin, streptomycin, and amphotericin (PSA). C6/36 mosquito cells from *Aedes albopictus* were cultured at 28°C in a 25-cm^2^ flask containing L-15 medium supplemented with 1% tryptose phosphate broth and 10% FBS and PSA. DENV-2 (NGC strain) and YFV (17DD vaccine strain) were amplified in C6/36 cells and titrated by the classic plaque assay with Vero E6 cells. The results are reported as plaque-forming units per milliliter (PFU/mL).

### Cytotoxicity of the samples

The cytotoxicity of Vero E6 cells treated with root extract and saponins was determined by MTT (3-(4,5-dimethylthiazol-2-yl)-2,5-diphenyltetrazolium bromide, Sigma-Aldrich, Germany) assay (20). Viable cells convert MTT into a purple-colored formazan product with a maximum absorbance of ∼570 nm, while dead cells fail to show this colorization. Briefly, confluent quadruplicate Vero E6 cell monolayers grown in 96-well plates were exposed to two-fold serial dilutions of each sample (L15 medium was used as the diluting solution). The cells were incubated for seven days at 37°C. Then, 50 μL of L-15 containing MTT (final concentration of 1 mg/mL) was added to each well. After 4 h of incubation at 37°C, the supernatant was removed, and 100 μL of DMSO was added to each well to solubilize the formazan crystals. After shaking, the absorbance was measured at 540 nm. The absorbance of the cells treated with the samples was compared with the absorbance of untreated cells to determine the percentage of viable cells. The concentration of each compound that reduced cell viability by 50% (cytotoxic concentration (CC_50_)) was determined by nonlinear regression analysis. The cytotoxicity induced by DMSO and ethanol, which were used to dissolve the samples, was also determined.

### Viral inhibition assay

The viral inhibition activity was evaluated by the plaque reduction assay using a non-cytotoxic concentration of the ethanolic extract of *S. sisymbriifolium* roots. Virucidal, pretreatment, and post-infection assays with DENV-2 (NGC strain) were performed as described previously (21). Briefly, for the virucidal assay, 10-fold serial dilutions of the extract were incubated with ∼50 PFU of DENV-2 for 1 h at 37°C. Each mixture was used to infect a monolayer of VERO E6 cells (2×10^5^ cells) by incubation a 37°C for 1 h. The supernatant was removed, and 1 mL of semisolid overlay medium, containing L-15 supplemented with 2% FBS and 0.9% carboxymethylcellulose, was added to the cells. After seven days of incubation at 37°C, the overlay medium was removed, and the cells were fixed and stained with naphthol blue-black in 5% acetic acid for the plaque lysis assay. In the pretreatment assay, the cells were treated with 2- or 10-fold serial dilutions of the extract for 3 h at 37°C. Then, the supernatant was removed, and the cells were infected with 400 µL of L-15 containing ∼50 PFU DENV-2 and incubated for 1 h at 37°C. The supernatant was removed, and the semisolid overlay medium was added to the cells, which were incubated for 7 days at 37°C. The number of lysed plaques was counted as mentioned above. In the post-infection assay, the cells were infected with ∼50 PFU in 400 mL of L-15 and incubated for 1 h at 37°C. The supernatant was removed, and 10-fold serial dilutions of the extract in 1 mL of semisolid overlay medium were added to the cells, which were incubated at 37°C for 7 days. The number of lysed plaques was counted as mentioned above. The number of plaques among the cells treated with the extract was compared with the number of plaques among the untreated cells to determine the percentage of plaque reduction. The 50% maximal effective concentration (EC_50_), which is the concentration of sample that reduces the number of plaques by 50%, was determined for each antiviral assay by nonlinear regression analysis. The EC_50_ values of both ethanol and DMSO were also determined. The inhibitory activity of the saponins isolated from the ethanolic extract of *S. sisymbriifolium* roots was evaluated in the virucidal assay using 2-fold serial dilutions.

### Analysis of the saponin virucidal effect by immunofluorescence assay

DENV-2 (∼50 PFU) was incubated with saponin samples S1 (102.33 µg/mL), S2 (104.71 µg/mL), and S3 (85.11 µg/mL) for 1 h at 37°C. Each mixture was used to infect a monolayer of VERO E6 cells (2×10^5^ cells) contained in a 16-well plate with a glass cover (CultureWell chambered coverglass, Invitrogen, USA) and incubated at 37°C for 1 h. The supernatant was removed, and 100 µL of semisolid overlay medium containing L-15 supplemented with 2% FBS and 0.9% carboxymethylcellulose was added to the cells. After four days of incubation at 37°C, the overlay medium was removed, and the cells were fixed and stained with 5% formaldehyde for 15 min at room temperature. The cells were washed with phosphate-buffered saline (PBS) and permeabilized with 50 µL of 0.5% Triton. The cells were washed again and blocked with 50 µL of 3% bovine albumin for 30 min at 37°C. A pool of hyper-immune sera from mice infected with DENV-1, DENV-2, DENV-3, or DENV-4 (20 µL of a 1:100 diluted pool) was incubated with the cells for 30 min at 37°C. The cells were washed and incubated for 30 min with 20 µL of anti-mouse IgG (1:100 dilution) conjugated with Cy3 (Sigma, USA) at 37°C. The cells were washed and incubated with 20 µL of 4′,6-diamidino-2-phenylindole dihydrochloride (DAPI, Invitrogen) for nuclei staining. The cells were visualized using a confocal microscope (Leica Microsystems TCS SP8, Germany), and the images were acquired and processed using Leica LAS X software (Leica Microsystems).

### Statistical analysis

The EC_50_ and CC_50_ values of the compounds were determined by nonlinear regression analysis using the GraphPad Prism for Windows, Version 6 (GraphPad Software Inc., USA) and reported as means±SE of quadruplicate assays. The number of Vero E6 cells infected with the untreated DENV-2 was compared with the number of Vero E6 cells infected with the saponin-treated DENV-2 and analyzed by Fisher's exact test with a 95% confidence interval. A P-value <0.05 indicated a significant difference between groups. GraphPad Prism was used for these analyses.

## Results

### Cytotoxicity of the ethanolic extract of *S. sisymbriifolium* roots

**Figure 1:**
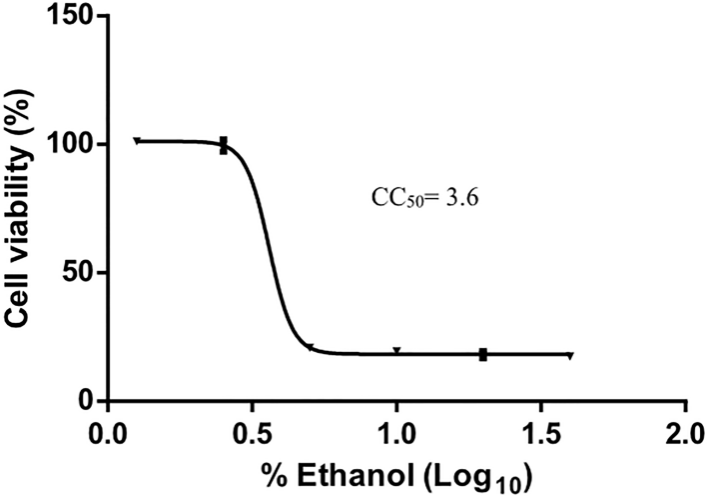
Cytotoxicity of ethanol. Vero E6 cells grown in a 24-well plate were treated with 2-fold serial dilutions of ethanol (starting concentration: 40%) for 7 days. Cell viability was evaluated by MTT assay. The cytotoxic concentration (CC_50_) value of the ethanol was determined by nonlinear regression analysis. The data are reported as means±SE of quadruplicate wells.

The ethanolic extract of *S. sisymbriifolium* roots was dissolved in ethanol; therefore, we first analyzed the cytotoxicity of ethanol. Vero E6 cells were treated with two-fold serial dilutions of ethanol, and the cytotoxicity was determined by MTT assay. A nonlinear regression analysis of cell viability showed that the CC_50_ value of ethanol was 3.6% (Figure 1).

The root extract (50 mg/mL of ethanol) was diluted 25 times with L15 medium to reduce the concentration of ethanol to be approximately the same as its CC_50_ value (final concentration: 2 mg/mL of extract and 4% of ethanol). Two-fold serial dilutions of this solution were used in MTT assays to determine the cytotoxicity of the extract in Vero E6 cells. The ethanol extract of *S. sisymbriifolium* roots showed no cytotoxicity in Vero E6 cells at the highest tested concentration (2 mg/mL).

### Inhibitory activity of the ethanolic extract of *S. sisymbriifolium* roots against DENV-2

**Figure 2:**
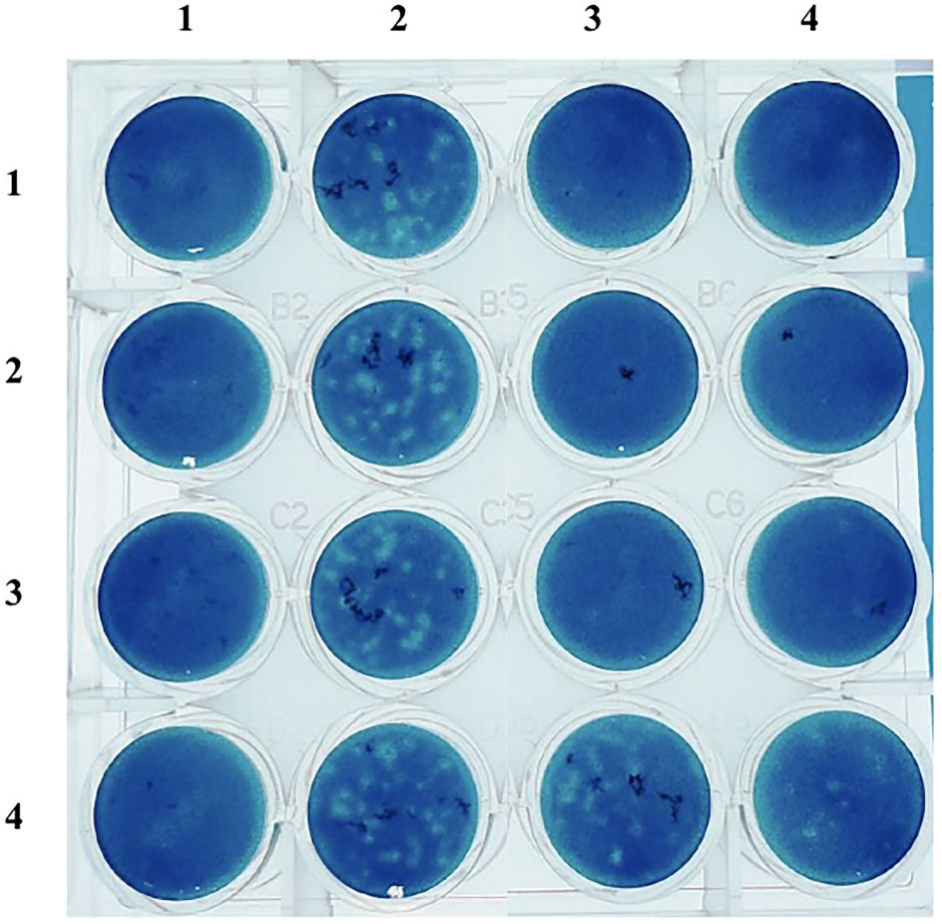
Virucidal assay of the ethanolic extract of *Solanum sisymbriifolium* roots against dengue virus (DENV-2). DENV-2 (∼50 PFU) was treated with 10-fold serial dilutions of the root extract and used to infect duplicate Vero E6 cell cultures grown in 24-well plates for 7 days. The extract concentrations used in the assay are as follows: in columns 3 and 4, line 1: 2000 µg/mL; line 2: 200 µg/mL; line 3: 20 µg/mL; and line 4, 2 µg/mL. Uninfected cells (column 1) and cells infected with untreated DENV-2 (column 2) were used as controls.

The inhibitory activity of the ethanolic extract of *S. sisymbriifolium* roots against DENV was examined using three different strategies: a virucidal assay to analyze the direct effects of the extract on the virus particles; pretreatment of cells to examine the resistance against viral infection conferred by the extract; and post-infection of cells to examine the influence of the extract on the virus multiplication cycle after penetration. We used the DENV-2 as a prototype virus of the DENV group to perform the antiviral assays. Initially, to determine the inhibitory activity of the ethanolic extract of *S. sisymbriifolium* roots against DENV-2, a pilot assay was performed for each antiviral strategy. The virus or Vero E6 cells, depending on the assay, were treated with 10-fold serial dilutions of the root extract (starting concentration: 2000 µg/mL). The root extract showed a dose-dependent inhibitory effect on DENV-2 multiplication in Vero E6 cells only in the virucidal assay (Figure 2). A clear inhibitory effect was observed with extract concentrations as high as 20 µg/mL.

**Figure 3:**
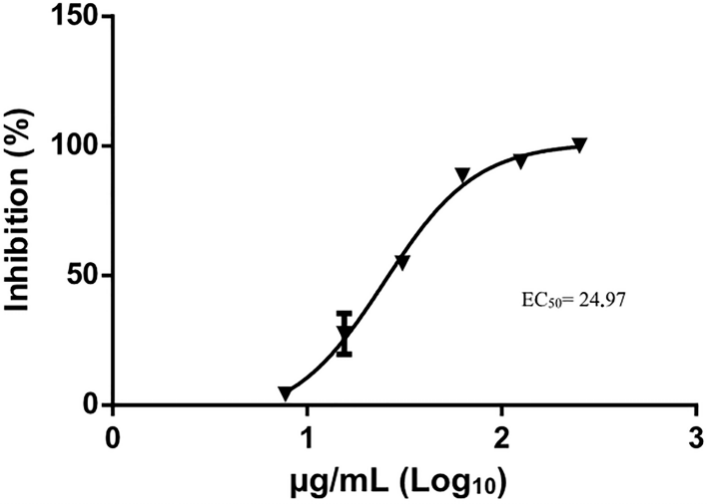
Plaque inhibition analysis of the ethanolic extract of *S. sisymbriifolium* roots in the virucidal assay. Dengue virus (DENV-2) was treated with 2-fold serial dilutions of the root extract for 1 h. Treated DENV-2 was used to inoculate monolayers of Vero E6 growth in a 24-well plate, which were then incubated for 7 days. The number of plaques in the wells with cells infected with the treated virus was compared with the number of plaques in the wells with cells infected with the untreated virus to determine the percentage of plaque reduction. The 50% maximal effective concentration (EC_50_) value of the extract was determined by nonlinear regression analysis. The data are reported as means±SE of quadruplicate wells.

To determine the EC_50_ of the ethanolic extract of *S. sisymbriifolium* roots in the virucidal assay, DENV-2 was treated with 2-fold serial dilutions of the extract and then used to inoculate Vero E6 cell monolayers for 7 days. The number of plaques was counted and compared with the number of plaques observed in the untreated cells infected with virus to calculate the percentage of cells with inhibited infection. A nonlinear regression analysis showed that the ethanolic extract of *S. sisymbriifolium* roots had an EC_50_ of 24.97 µg/mL (Figure 3). At this concentration of the extract, the ethanol concentration was less than 0.5% and showed no virucidal activity against DENV-2 (Supplementary Table S1), confirming that the inhibitory activity was due to the roots and not to the ethanol contained in the solution.

### Virucidal potential of the saponins isolated from *S. sisymbriifolium* roots

**Figure 4:**
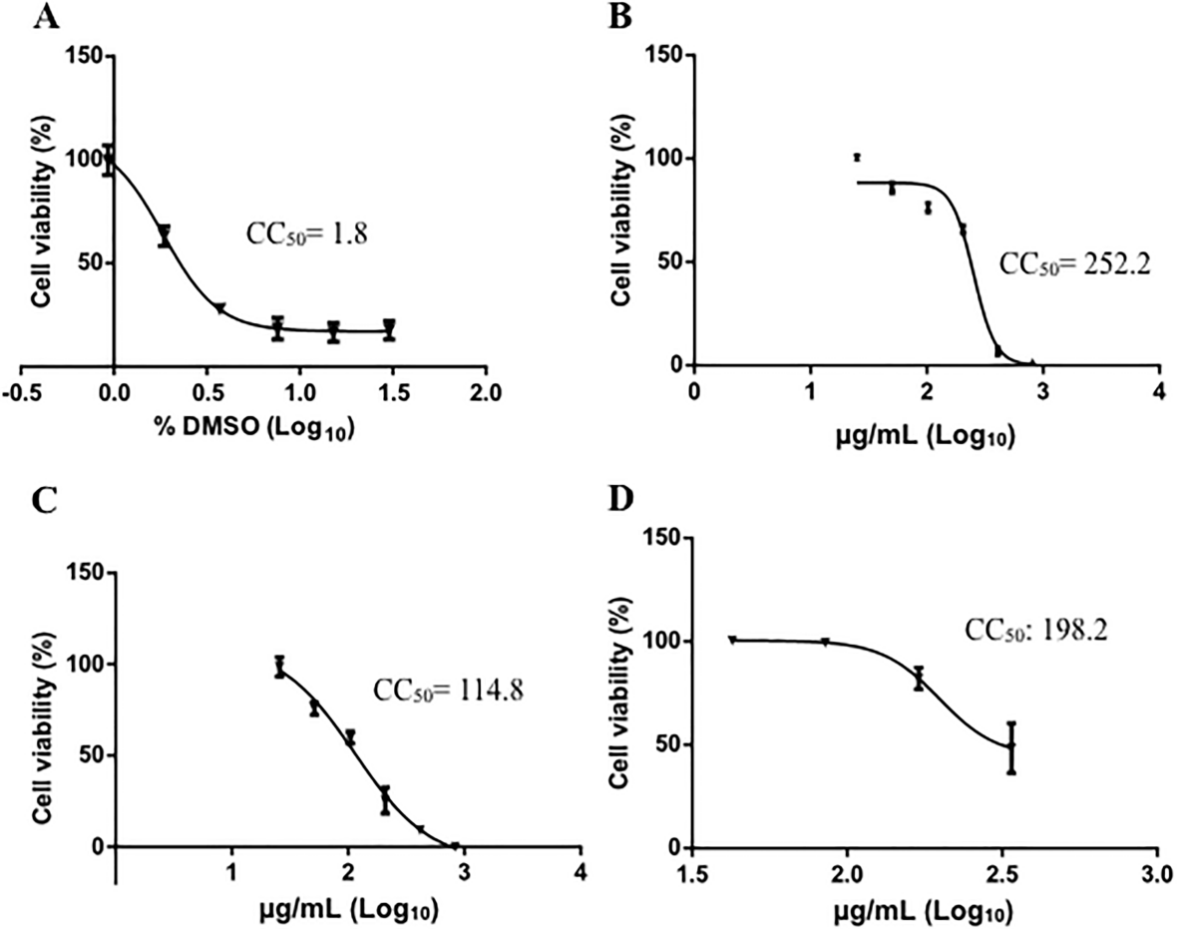
Cytotoxicity of the saponins and DMSO. Vero E6 cells grown in a 24-well plate were treated with 2-fold serial dilutions of DMSO (**A**) and saponins S1 (**B**), S2 (**C**), and S3 (**D**) and then incubated for 7 days. Cell viability was evaluated by MTT assay. The cytotoxic concentration (CC_50_) values of DMSO and saponins were determined by nonlinear regression analysis. The data are reported as means±SE of quadruplicate wells.

To investigate whether saponins were critical for the inhibitory effect of the extract, three saponins were purified from the roots of *S. sisymbriifolium* and analyzed by virucidal assay. Initially, the cytotoxicity of the saponins and DMSO, which was used to dissolve the saponins, was analyzed (Figure 4).

**Figure 5:**
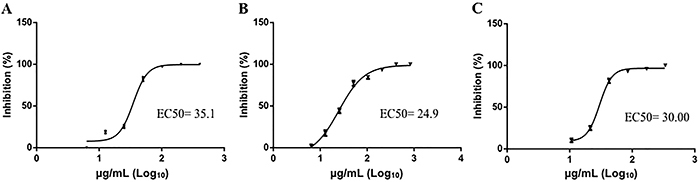
Plaque inhibition analysis of saponins against dengue virus (DENV-2) by virucidal assay. The virus was treated with 2-fold serial dilutions of the saponins S1 (**A**), S2 (**B**), and S3 (**C**) isolated from the roots of *S. sisymbriifolium* for 1 h. The treated DENV-2 was used to infect monolayers of Vero E6 cells growing in a 24-well plate for 7 days. The number of plaques in the wells with cells infected with the treated virus was compared with the number of plaques in the wells with cells infected with the untreated virus to determine the percentage of plaque reduction. The 50% maximal effective concentration (EC_50_) value of the saponins was determined by nonlinear regression analysis. The data are reported as means±SE of quadruplicate wells.

Non-cytotoxic concentrations of saponin samples and DMSO were used in the virucidal assay, and the results showed that all of the isolated saponins exerted an inhibitory effect against DENV-2 (Figure 5). DMSO did not show a virucidal effect against DENV-2 below a concentration of 1%, which was the highest concentration of DMSO used to solubilize the saponins (Supplementary Table S2).

**Figure 6:**
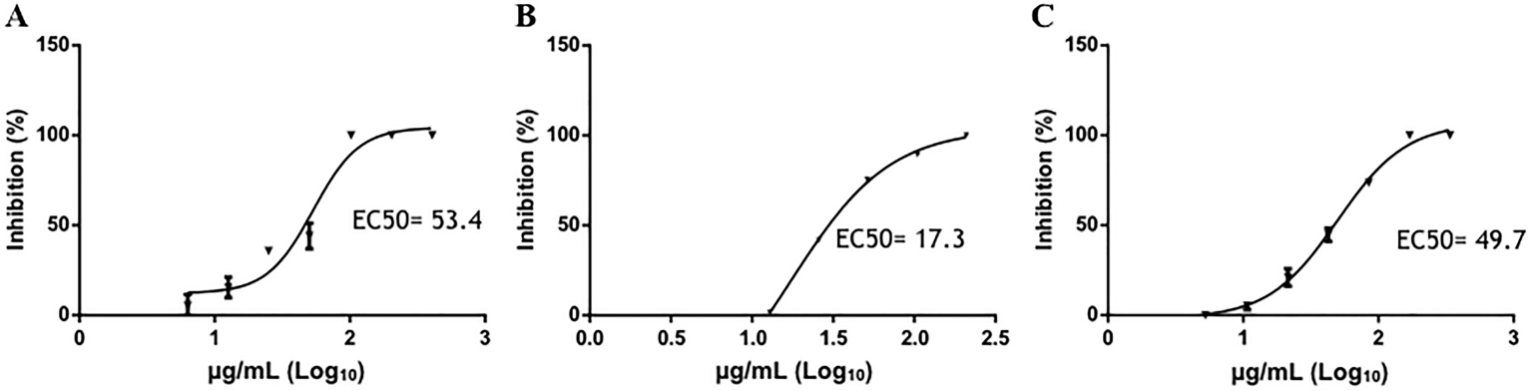
Plaque inhibition analysis of saponins against yellow fever virus in the virucidal assay. The virus was treated with 2-fold serial dilutions of saponins S1 (**A**), S2 (**B**), and S3 (**C**) isolated from the ethanolic extract of *S. sisymbriifolium* roots for 1 h. The treated yellow fever virus was used to infect monolayers of Vero E6 cells growing in a 24-well plate for 7 days. The number of plaques in the wells with cells infected with the treated virus was compared with the number of plaques in the wells with cells infected with the untreated virus to determine the percentage of plaque reduction. The 50% maximal effective concentration (EC_50_ value) of the saponins was determined by nonlinear regression analysis. The data are reported as means±SE of quadruplicate wells.

To analyze whether the saponins can inhibit other flaviviruses, we analyzed their effect against YFV, the prototype virus of the *Flavivirus* genus. All saponins showed virucidal activity against YFV (Figure 6). DMSO did not show a virucidal effect against YFV at a concentration less than 1% (Supplementary Table S3). Moreover, all saponins showed good selectivity indices (SI, CC_50_/EC_50_) against DENV-2 and YFV (Table 1).

**Table 1:** Virucidal activity of the saponins isolated from the roots of *S. sisymbriifolium*.

Saponins	VERO E6	Dengue virus type 2		Yellow fever virus	
	CC_50_ (µg/mL)	EC_50_ (µg/mL)	SI	EC_50_ (µg/mL)	SI
S1	252.2	35.1	7.2	53.4	4.7
S2	114.8	24.9	4.6	17.3	6.6
S3	198.2	30.0	6.6	49.7	4.0

CC_50_: concentration of saponin that reduced cell viability by 50%; EC_50_: concentration of saponin that reduced in 50% the number of plaques of lysis; SI: selectivity index; S1: saponin 1; S2: saponin 2; S3: saponin 3.

### Analysis of the virucidal activity of saponins by immunofluorescence staining assay

**Figure 7:**
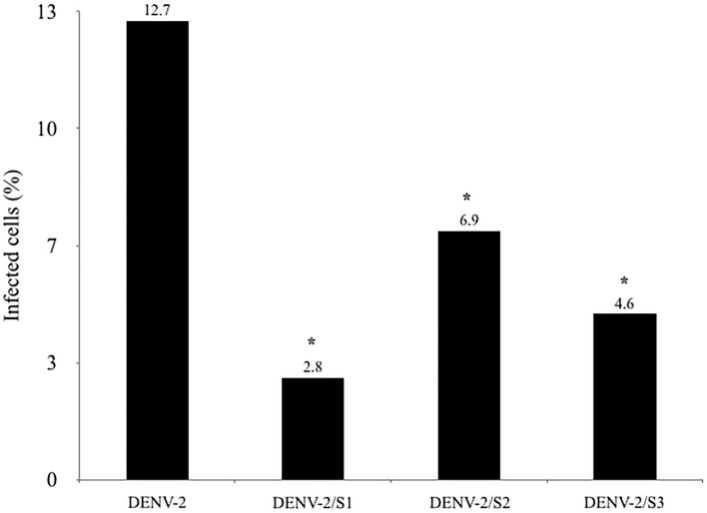
Quantification of the number of Vero E6 cells infected with dengue virus (DENV-2). Vero E6 cells were infected for four days with DENV-2 treated with saponins S1, S2, and S3, and then, the number of infected cells was determined by an immunostaining assay. Uninfected Vero E6 cells and cells infected with untreated virus were used as controls. The number of infected cells after infection with DENV-2 treated with saponins was compared with the number of cells infected with the untreated virus in 20 random fields to determine the rate of inhibition of viral infection. *P<0.001 between cells infected with treated and untreated virus (Fisher's exact test).

The virucidal activity of the saponins against DENV-2 was further analyzed by an immunofluorescence staining assay. A significant reduction in Vero E6 cells infected with DENV-2 was observed when the virus was treated with the saponins (Figure 7).

**Figure 8:**
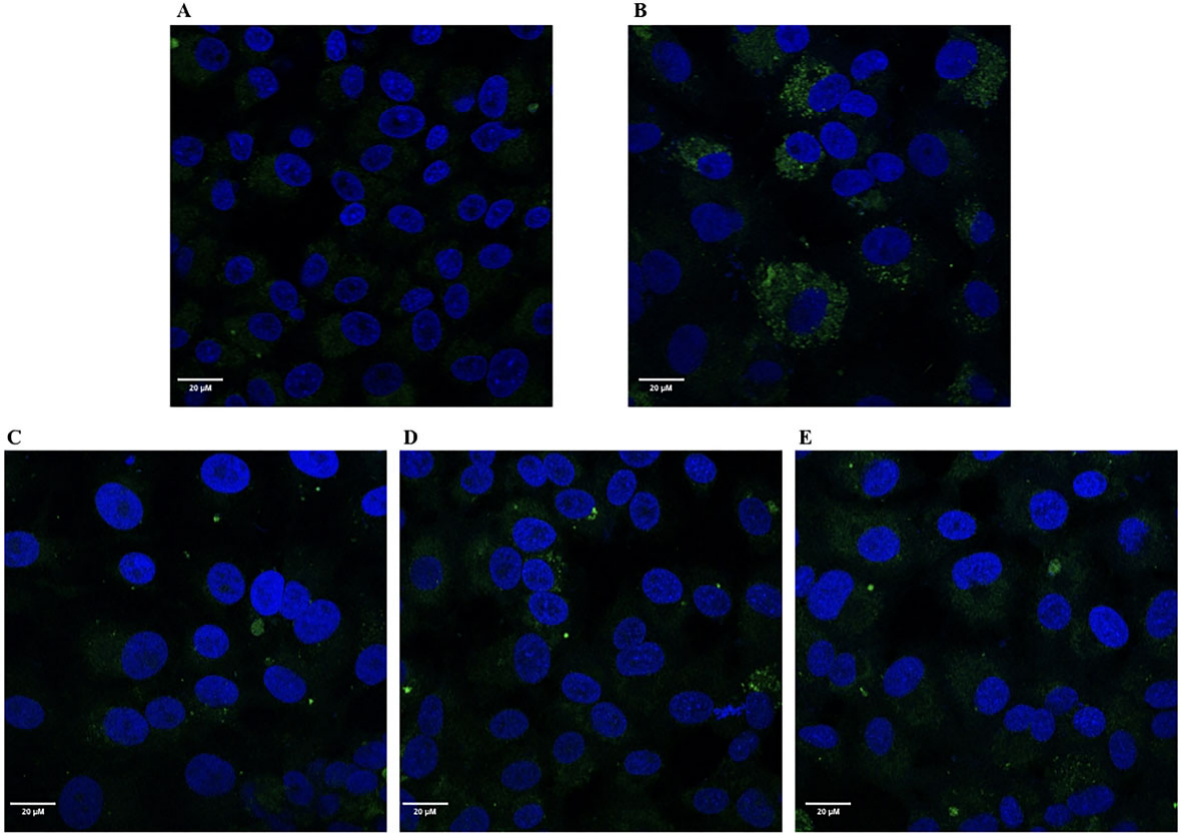
Confocal microscopy images of Vero E6 cells after immunofluorescence staining. **A**, Uninfected Vero E6 cells. **B**, Vero E6 cells infected with untreated dengue virus (DENV-2). Vero E6 cells infected with DENV-2 treated with saponin S1 (**C**), saponin S2 (**D**), and saponin S3 (**E**). DNA was counterstained with DAPI (blue fluorescence). Scale bar 20 μm.

Figure 8 shows representative images of the viral inhibitory activity of saponins in the immunofluorescence-staining assay.

### Identification of the saponins

Saponin S2 was identified as nuatigenin-3-O-β-chacotriose (nuatigenoside) through a comparison of its spectroscopic data with those in the literature (22). Amorphous powder; m/z: 884 [M - H]^-^; ^1^H-NMR (pyridine-d5): δ 6.39 (1H, br s, H-1-), 6.85 (1H, br s, H-1′-), 5.31 (1H, br d, J=5.1 Hz, H-6), 4.96 (1H, d, J=7.8 Hz, H-1′), 1.77 (3H, d, J=6.2 Hz, H-6-), 1.64 (3H, d, J=6.2 Hz, H-6′-), 1.40 (3H, s, H-27), 1.08 (3H, d, J=6.9 Hz, H-21), 1.05 (3H, s, H-19), 0.81 (3H, s, H-18). The identification of the two other saponins (S1 and S3) is ongoing, but the spectroscopic data indicate that they are structurally related to nuatigenoside.

## Discussion

The plants of the *Solanum* genus have been employed as anticancer and antiviral agents in many countries since ancient times (23,24). In the present study, we found that the ethanolic extract of *S. sisymbriifolium* roots showed inhibitory activity against DENV-2 without an evident cytotoxic effect in Vero E6 cells. Similarly, antiviral activities against other viruses, such as human herpesvirus type 1, vaccinia virus, and hepatitis C virus has been found with other plants of the *Solanum* genus (25–27). Among the compounds isolated from plants of the *Solanum* genus, glycosteroidal saponins isolated from *S. paniculatum* and *S. torvum* have been shown to exert antiviral activity against human herpesvirus type 1 and vaccinia virus (25,27). In addition, triterpene saponins isolated from the plants of other genera have shown antiviral activities against human respiratory syncytial virus, human immunodeficiency virus, human herpes simplex type 1 and 2 viruses, human rhinovirus, tobacco mosaic virus, hepatitis B virus (HBV), and influenza virus (28–33). Similarly, uralsaponins isolated from *Glycyrrhiza uralensis* and saikosaponin A derived from the root of *Bupleurum chinense* DC have also shown anti-influenza virus (H1N1) activity (32,34).

In the present study, three saponins (saponins S1, S2, and S3) were isolated from the ethanolic extract of *S. sisymbriifolium* roots. Saponin S2 was identified as nuatigenoside, which was first isolated from the bulbs of *Triteleia lactea* (22) and later from the roots of *S. sisymbriifolium* (14). Saponin S2 has shown antihypertensive activity (35) and inhibitory activity against cyclic AMP phosphodiesterase (22), but to the best of our knowledge, this is the first study describing its antiviral activity. The four distinct serotypes of DENV are antigenically related, but immunologically distinct. Although they show different serological profiles, they share similar structure and biology. Therefore, it is highly likely that the virucidal effect of the plant extract and compounds against DENV-2 found in this study are similar to that of other DENV serotypes.

The three saponins isolated in this study showed inhibitory activity against DENV-2 and YFV, which is the prototype virus of the *Flavivirus* genus, suggesting their potential antiviral effect against other members of this genus. Although a low SI was found for the isolated saponins, these saponins are good natural starting materials for synthetic modification and drug discovery due to their natural abundance and high availability (36,37). For example, synthetic saponin derivatives have shown inhibitory activity against H5N1, an influenza virus (38). Finally, saponins may not be the only compounds with viral inhibitory activity in the ethanolic root extract of *S. sisymbriifolium*; further studies are needed to identify other molecules with antiviral activity in this plant.

In summary, we have found a highly inhibitory effect of an ethanolic extract of *S. sisymbriifolium* roots against DENV-2. Saponins isolated from this root extract showed inhibitory activity against DENV-2 and YFV. The results obtained in this study and data available in the literature suggest the therapeutic potential of these saponins, which may be lead compounds for the development of antiviral agents.

## Supplementary Material

Click here to view [pdf].

## References

[1] Bhatt S, Gething PW, Brady OJ, Messina JP, Farlow AW, Moyes CL (2013). The global distribution and burden of dengue. Nature.

[2] Brady OJ, Gething PW, Bhatt S, Messina JP, Brownstein JS, Hoen AG (2012). Refining the global spatial limits of dengue virus transmission by evidence-based consensus. PLoS Negl Trop Dis.

[3] WHO (World Health Organization) (2009). Dengue: guidelines for diagnosis, treatment, prevention and control: new edition.

[4] WHO (World Health Organization) (2017). GACVS Statement on Dengvaxia® (CYD-TDV).

[5] Kadir SLA, Yaakob H, Zulkifli RM (2013). Potential anti-dengue medicinal plants: a review. J Nat Med.

[B06] Muller VD, Soares RO, dos Santos NN, Trabuco AC, Cintra AC, Figueiredo LT (2014). Phospholipase A2 isolated from the venom of *Crotalus durissus terrificus* inactivates dengue virus and other enveloped viruses by disrupting the viral envelope. PLoS One.

[7] Rothan HA, Buckle MJ, Ammar YA, Mohammadjavad P, Shatrah O, Noorsaadah AR (2013). Study the antiviral activity of some derivatives of tetracycline and non-steroid anti inflammatory drugs towards dengue virus. Trop Biomed.

[8] Staples JE, Monath TP (2008). Yellow fever: 100 years of discovery. JAMA.

[9] Barrett AD, Monath TP (2003). Epidemiology and ecology of yellow fever virus. Adv Virus Res.

[10] Afroz M, Akter S, Ahmed A, Rouf R, Shilpi JA, Tiralongo E (2020). Ethnobotany and antimicrobial peptides from plants of the solanaceae family: an update and future prospects. Front Pharmacol.

[11] Shonle I, Bergelson J (2000). Evolutionary ecology of the tropane alkaloids of *Datura stramonium* L. (Solanaceae). Evolution.

[12] Bongo GN, Mutunda CM, Inkoto CL, Mbadiko CM, Lengbiye E, Dorothée TD (2020). Review on ethno-botany, virucidal activity, phytochemistry and toxicology of solanumgenus: potential bio-resources for the therapeutic management of covid-19. Eur J Nutrit Food Safety.

[13] Torres DG (2013). Catalogo de plantas medicinales (y alimenticias y útiles) usadas en el Paraguay [in Spanish].. AsuncionL Servi Libro.

[14] Ibarrola DA, Hellión-lbarrola MC, Montalbetti Y, Heinichen O, Alvarenga N, Figueredo A (2000). Isolation of hypotensive compounds from *Solanum sisymbriifolium* Lam. J Ethnopharmacol.

[15] Ibarrola DA, Ibarrola MH, Vera C, Montalbetti Y, Ferro EA (1996). Hypotensive effect of crude root extract of *Solanum sisymbriifolium* (Solanaceae) in normo- and hypertensive rats. J Ethnopharmacol.

[16] Filipov A (1994). Medicinal plants of the Pilagá of central Chaco. J Ethnopharmacol.

[17] Chakravarty AK, Mukhopadhyay S, Saha S, Pakrashi SC (1996). A neolignan and sterols in fruits of *Solanum sisymbrifolium*. Phytochemistry.

[B18] Chand R, Kumar S, Sharma AK, Srivastava L (1995). Variation of solasodine in *Solanum sisymbriifolium* and *Solanum xanthocarpum* with plant growth and development. Indian Drugs.

[19] Ferro EA, Alvarenga NL, Ibarrola DA, Hellión-Ibarrola MC, Ravelo AG (2005). A new steroidal saponin from *Solanum sisymbriifolium* roots. Fitoterapia.

[20] Mosmann T (1983). Rapid colorimetric assay for cellular growth and survival: application to proliferation and cytotoxicity assays. J Immunol Methods.

[21] Muller VDM, Russo RR, Cintra ACO, Sartim MA, Alves-Paiva RM, Figueiredo LTM (2012). Crotoxin and phospholipases A2 from *Crotalus durissus terrificus* showed antiviral activity against dengue and yellow fever viruses. Toxicon.

[22] Mimaki Y, Nakamura O, Sashida Y, Nikaido T, Ohmoto T (1995). Steroidal saponins from the bulbs of *Triteleia lactea* and their inhibitory activity on cyclic AMP phosphodiesterase. Phytochemistry.

[23] Ikeda T, Tsumagari H, Honbu T, Nohara T (2003). Cytotoxic activity of steroidal glycosides from solanum plants. Biol Pharm Bull.

[24] Nakamura T, Komori C, Lee Y, Hashimoto F, Yahara S, Nohara T (1996). Cytotoxic activities of solanum steroidal glycosides. Biol Pharm Bull.

[25] Arthan D, Svasti J, Kittakoop P, Pittayakhachonwut D, Tanticharoen M, Thebtaranonth Y (2002). Antiviral isoflavonoid sulfate and steroidal glycosides from the fruits of *Solanum torvum*. Phytochemistry.

[B26] Javed T, Ashfaq UA, Riaz S, Rehman S, Riazuddin S (2011). In vitro antiviral activity of Solanum nigrum against Hepatitis C Virus. Virol J.

[27] Valadares YM, Brandão'a GC, Kroon EG, Filho JD, Oliveira AB, Braga FC (2009). Antiviral activity of *Solanum paniculatum* extract and constituents. Z Naturforsch C J Biosci.

[28] He Z, Qiao C, Han Q, Wang Y, Ye W, Xu H (2005). New triterpenoid saponins from the roots of *Platycodon grandiflorum*. Tetrahedron.

[B29] Lückemeyer DD, Müller VD, Moritz MI, Stoco PH, Schenkel EP, Barardi CR (2012). Effects of *Ilex paraguariensis* A. St. Hil. (yerba mate) on herpes simplex virus types 1 and 2 replication. Phytother Res.

[B30] Mair CE, Grienke U, Wilhelm A, Urban E, Zehl M, Schmidtke M (2018). Anti-influenza triterpene saponins from the bark of *Burkea africana*. J Nat Prod.

[B31] Rao GS, Sinsheimer JE (1974). Antiviral activity of triterpenoid saponins containing acylated beta-amyrin aglycones. J Pharm Sci.

[32] Song W, Si L, Ji S, Wang H, Fang XM, Yu LY (2014). Uralsaponins M-Y, antiviral triterpenoid saponins from the roots of *Glycyrrhiza uralensis*. J Nat Prod.

[33] Zhou M, Xu M, Ma XX, Zheng K, Yang K, Yang CR (2012). Antiviral triterpenoid saponins from the roots of *Ilex asprella*. Planta Med.

[34] Chen J, Duan M, Zhao Y, Ling F, Xiao K, Li Q (2015). Saikosaponin A inhibits influenza A virus replication and lung immunopathology. Oncotarget.

[35] Ibarrola DA, Hellión-Ibarrola MC, Montalbetti Y, Heinichen O, Campuzano MA, Kennedy ML (2011). Antihypertensive effect of nuatigenin-3-O-β-chacotriose from *Solanum sisymbriifolium* Lam. (Solanaceae) (ãuatî pytâ) in experimentally hypertensive (ARH+DOCA) rats under chronic administration. Phytomedicine.

[36] Lin YY, Chan SH, Juang YP, Hsiao HM, Guh JH, Liang PH (2018). Design, synthesis and cytotoxic activity of N-modified oleanolic saponins bearing A glucosamine. Eur J Med Chem.

[37] Wang YH, Yeh HW, Wang HW, Yu CC, Guh JH, Liu DZ (2013). Synthesis of a chlorogenin glycoside library using an orthogonal protecting group strategy. Carbohydr Res.

[38] Song G, Shen X, Li S, Li Y, Si H, Fan J (2016). Structure-activity relationships of 3-O-β-chacotriosyl oleanane-type triterpenoids as potential H5N1 entry inhibitors. Eur J Med Chem.

